# Intervention against the stigmatization of men with eating disorders in primary care (iSMEsH): Protocol for a randomized mixed-methods evaluation trial

**DOI:** 10.1371/journal.pone.0333997

**Published:** 2025-10-09

**Authors:** Martin S. Lehe, Georg Halbeisen, Georgios Paslakis

**Affiliations:** 1 University Clinic for Psychosomatic Medicine and Psychotherapy, Medical Faculty, Campus East-Westphalia, Ruhr-University Bochum, Lübbecke, Germany; 2 Department of Clinical Psychology and Psychotherapy, University of Bamberg, Bamberg, Germany; PLOS: Public Library of Science, UNITED KINGDOM OF GREAT BRITAIN AND NORTHERN IRELAND

## Abstract

**Introduction:**

Eating disorders (EDs) are a significant public health concern, yet men remain underrepresented in research and care, partly due to stigmatization. This stigmatization contributes to reduced help-seeking and recognition of ED symptoms in men. To address this, targeted interventions for healthcare professionals are needed. The iSMEsH intervention aims to sensitize general practitioners (GPs) in Germany to EDs in men, impart relevant knowledge and skills, and counter the perception of EDs as “women’s diseases”.

**Methods:**

We will evaluate the iSMEsH intervention regarding its effects on biased attitudes, knowledge, and self-efficacy among GPs. A sequential explanatory mixed-methods design (QUAN → qual) will be applied in three steps: (1) pre-implementation focus groups and a panel discussion with individuals with lived ED experience and GPs to design the intervention, (2) conduction of a randomized, wait-list controlled trial of the online training trial with GPs and medical students, and (3) post-implementation qualitative interviews with GPs. Quantitative data (step 2) will be analyzed using mixed-measures ANOVAs and contrast analyses (per-protocol) as well as corresponding 2 × 3 linear mixed models with fixed and random effects (intention-to-treat). Qualitative data from step 3 will be analyzed using thematic analysis as outlined by Braun and Clarke (2006). Ethical approval was granted by the Ruhr-University Bochum Ethics Committee (AZ 2023−1106). Participants will provide written or digital informed consent.

**Discussion:**

The intervention seeks to reduce stigma against men with EDs in primary care by improving GPs’ and medical students’ knowledge, attitudes, and self-efficacy. Strengths include the comprehensive mixed-methods approach and involvement of people with lived experience. Limitations are reliance on self-report and short-/intermediate-term outcomes. If effective, iSMEsH may offer a foundation for future stigma-reduction strategies in healthcare.

## Introduction

### Background and rationale

Eating disorders (EDs) are a growing public health concern [1]. Their most common forms include Binge-eating disorder (uncontrollable binge eating episodes without compensatory behaviors), Bulimia nervosa (binge eating episodes followed by compensatory behaviors), Anorexia nervosa (restrictive eating leading to underweight), and other as well as subthreshold forms that do not eet the exact diagnostic criteria of the previously mentioned categories. People with EDs often have considerable health impairments and a significantly increased mortality risk compared to the general population [2]. Due to the frequent occurrence in adolescence, long duration of illness and repeated inpatient stays, the treatment costs are substantial [3].

Although women constitute the majority of ED cases across age groups, prevalence estimates suggest that men could account for every fourth case [4]. Men, however, remain underrepresented in ED research and care, for example, with one man with Anorexia nervosa or Bulimia nervosa for every 10–20 women in specialized treatment facilities [5,6]. One of the many probable reasons for men’s underrepresentation may be the so-called “double stigmatization” [7]: Men conceal having an ED not only because they are ashamed of having a mental disorder itself, but also because having an ED, which is broadly considered a “women’s disease”, conflicts with traditional gender roles and masculine identity norms. Internalizing such stereotypes (in the sense of self-stigmatization) makes recognizing and disclosing ED symptoms in medical and therapeutic contexts unlikely, and has been shown to reduce help-seeking intentions [8,9].

Men’s self-imposed stigmatization makes it essential for physicians to regularly ask about EDs in men, address them, if suspected, and refer men to appropriate treatment. At the same time, the stereotype of EDs as a “women’s disease” also shapes the views and behaviors of medical professionals [10]. A systematic review by our group of the experiences of men with EDs evidenced that symptoms often remain undetected, complaints are not being taken seriously, and the willingness to provide care by healthcare professionals is low [11]. Treatment access is further complicated by the fact that ED symptoms and body ideals often differ between men and women (e.g., striving for high muscle mass vs. a slim body shape) [12]. The question of how stereotypes and stigmatization in healthcare professionals and medical care for men with EDs can be combated remains.

Previous studies suggest that combined approaches of providing information and contact with those affected pose an effective anti-stigma strategy in working with healthcare professionals, like, for example, in the Canadian Opening Minds Anti-Stigma Initiative [13,14]. Similar approaches have been explored in the specific context of EDs [15]. Based on research into illness narratives, Bartel and Baker [16] developed a training course as an online intervention for UK general practitioners (GPs), which combined perspectives from men with EDs and educational materials via animated videos and art-based interventions. In Germany, there are currently campaigns aimed at informing men with EDs [17], but there are no interventions that explicitly address GPs, despite their key role as gatekeepers in the German healthcare system [18]. Thus, there is a continuing need to act against the stigmatization of men with EDs among GPs in Germany.

### Objectives

This project aims to sensitize GPs in primary care in Germany to the occurrence and manifestation of EDs in men, to impart knowledge and specific skills, and to act against the stigmatization of EDs as “women’s diseases”. Towards that end, we will develop, disseminate, and evaluate an intervention (in terms of implementation and efficacy) adapted for Germany, based on an existing online training course, “Eating disorders in boys and men for doctors”. Although a preliminary version of the training course has already been created by our group and made openly available [19], it remains unclear who uses the course, whether it is accepted and implemented as intended, and how effective the course is as an intervention against stigmatization. Thus, the objectives of the current project are (1) to revise and optimize the existing training materials for their use in the German healthcare system, to (2) systematically record and evaluate aspects of implementation according to the “Implementation Outcomes” framework model [20] (i.e., acceptability, feasibility, appropriateness, adoption, fidelity, penetration, sustainability), and to (3) assess the effects of the intervention on reducing the identified sources of stigmatization (knowledge deficits, biased attitudes, low counselling and action competence). In this regard, we hypothesized that participation in the intervention will lead to individual increases in knowledge, decreases in stigmatizing attitudes, and increases in self-efficacy.

### Trial design

**Fig 1:**
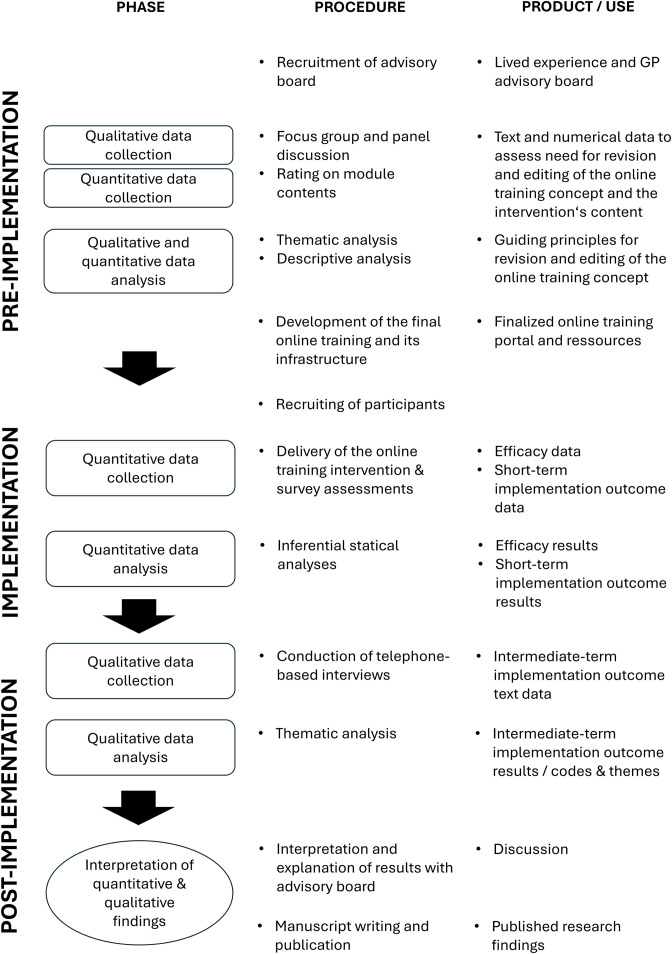
Visual model depicting the mixed-methods trial design. GP = General practitioner.

This study adopts a sequential exploratory mixed-methods design (QUAN → qual) guided by pragmatism principles (see Fig 1) [21]: In a pre-implementation stage (1), we will use both qualitative and quantitative data from a focus group, panel discussion, and rating procedure with both individuals with lived experience of EDs and GPs to guide the editing and adaptation of the intervention. In the implementation stage (2), we will deliver the online training in a randomized, wait-list controlled multiple group design to GPs and medical students in their clinical training year (“Praktisches Jahr”, last year of medical school), assessing quantitative endpoints of implementation and efficacy (‘QUAN’) before and after the participation. In a final post-implementation stage (3), we will then conduct telephone-based qualitative interviews (‘qual’) with GPs who participated in the online training assessing further implementation outcomes.

## Materials and methods

### Study setting and participant recruitment

Study participants will be recruited nationwide, targeting individuals with lived experience of EDs, GPs, and medical students. Recruitment will be conducted through offline and online methods, including leaflets, personal recommendations and mass emailings. We will perform extensive web-scraping of contact registers of medical professional organizations and apply for access to the federal register of physicians for a nation-wide probability sample of GPs. Medical students in their clinical training year will be recruited through local university networks and mass emailing via clinical training year coordinators at medical faculties across all German universities.

Individuals with lived experience will be rewarded 100 € for participating in the focus group, and 75 € for each attended advisory board meeting (for further details, see section on Patient and Public Involvement). GPs participating in the online training will receive 25 € reimbursement upon completion as well as 1 continuing medical education (CME) credit point, and another 50 € for participating in the telephone-based qualitative interview. Medical students will be eligible to register for a prize draw to win online vouchers worth 375 € in total. All participants will receive a certificate upon successful completion of the online training.

### Eligibility criteria

Focus group participants with lived experience of an ED must (i) identify as male, (ii) be aged 18 years or older, (iii) able and willing to provide written informed consent, and (iv) endorse having received a current or past ED diagnosis. Participants with lived experience of an ED will be excluded if, during the preliminary meeting, they exhibit significant distress related to their ED or to discussing it. The focus group will further include GPs, who are eligible if they are (i) of legal age and capacity and (ii) regularly treat men in their healthcare institution.

To be included in the online training intervention, participants (i) must be aged 18 years or older, (ii) work in the German healthcare system as a GP, or study medicine at a German university in their clinical training year (“Praktisches Jahr”), (iii) are involved in the medical treatment of men in their current position, and (iv) be able and willing to provide written informed consent. Individuals not meeting these criteria will be excluded from the study.

### Intervention

Eligible participants will be randomized to the intervention (i.e., immediate access to the 14-day self-paced online training period) or to a wait-list control group (i.e., 14-day delay before online training access) following a 1:1 ratio using a random number generation procedure implemented in Microsoft Excel. Participants will be blinded as per the different conditions. The intervention will consist of an on-demand online training which will be implemented in a Moodle learning management system to ensure better integration into the everyday work of clinicians through easy accessibility and self-paced learning. Such formats are common in the field [22] and follow recommendations for engaging online trainings [23,24]. For reasons of compatibility with everyday working life, we also deliberately opted for short, separate modules after carefully weighing up the advantages and disadvantages. The six modules are presented as learning videos with presentation slides, demonstrational dialogues, reflexive exercises and supporting downloadable in-depth resources, and should be completed online in 5–10 minutes each (corresponding to a total duration of 45–50 minutes), followed by interactive questions, ratings and quizzes. The module structure and corresponding contents are summarized in Table 1.

**Table 1:** iSMEsH Online Training Intervention Modules and Content.

Module #	Title	Content
1	Introduction to Eating Disorders	• Diagnostic criteria, clinical impressions and epidemiology of EDs in Boys and Men • Etiological Concepts • Promoting sensitivity to diversity • Implications of stigmatization as “second illness”
2	Symptoms and Diagnostics	• Clinical signs for suspected EDs on the psychological, social and physical level • Guiding questions for psychological and physical examination • Standardized diagnostic instruments (questionnaires, interviews) • Basic principles of somatic co-treatment • (Compulsive) hospital referral and stepped care
3	Communication Skills	• Recommendations for inclusive and non-discriminatory communication • Directly addressing stigma with patients • Basic principles and concepts of Motivational Interviewing • Perspective-taking exercise
4	Muscle Dysmorphia	• Diagnostic criteria, clinical impression and epidemiology of muscle dysmorphia as a subtype of body dysmorphic disorder • Role of physical exercise and substance abuse (e.g., anabolic steroids) • Masculinity ideals and male body image • Reflexive exercise on own internalized gender roles and masculinity concepts
5	Therapeutical, Social and Other Support Services	• Barriers to care for boys and men • Role of self- and public stigmatization and structural discrimination • Recommendations for adequate care • Introduction to medical, psychotherapeutic, social and other support services
6	Role and Support of Caregivers	• Inviting, inclusive and non-discriminatory communication with caregivers • Role of caregivers in the treatment process • Active support for caregivers

iSMEsH = Intervention Against the Stigmatization of Men with Eating Disorder in Primary Care; ED = Eating disorder.

The contents of the intervention are based on a social-cognitive and sociological understanding of ED-related stigma as a multi-facetted construct: (a) knowledge deficits (i.e., stereotypes), (b) biased attitudes (i.e., prejudices), and (c) behaviors (i.e., discrimination) [13]. Based on this understanding of stigma, the contents of the planned intervention thus combines demonstrably effective measures to reduce stigma [15], namely (a) thorough information to reduce knowledge deficits, (b) contact with the perspective of men with lived experience to reduce prejudice via patient narratives and case examples, and (c) the teaching of practical skills (e.g., via exemplary dialogues, hands-on recommendations and wordings for assessment and counseling) and the provision of resources to develop non-discriminatory counseling and treatment skills. The intervention will be established with extensive patient and public involvement, closely building up on the needs and narratives of up to ten men with ED experience and the recommendations of two GPs from a focus group prior to dissemination.

### Outcomes

The primary outcomes include the cognitive, affective and behavioral facets of ED-associated stigma and implementation quality. The cognitive aspects will be assessed with ten collaboratively developed single-choice questions to assess knowledge on EDs in boys and men; affective aspects will be assessed using the German version [25] of the Opening Minds Stigma Scale for Health Care Providers (OMS-HC) [26]; behavioral aspects will be assessed using the General Self-Efficacy Scale (GSE) [27]. Following Proctor et al. [20], implementation quality will be assessed along the dimensions of acceptability, feasibility, appropriateness, and adoption with self-developed rating questions; fidelity, penetration and sustainability will be assessed in a semi-structured interview by means of open-ended questions.

The secondary outcome includes stigma-related perceptions of EDs in men (Lehe et al., 2024).

Additional baseline variables considered potential covariates include sociodemographic features via the Diversity Minimal Item Set (DiMIS) [28] and self-developed items, study and job characteristics, professional medical background, professional attitude towards mental health, personal experiences with mental disorders and impairments, professional experiences with patients with EDs. We will additionally assess participants’ self-perceived identification with masculine and feminine gender roles at baseline through the Traditional Masculinity-Femininity Scale (TMF) [29] to assess potential moderating effects of gender role endorsement regarding anti-stigma effects.

### Participant timeline

**Fig 2:**
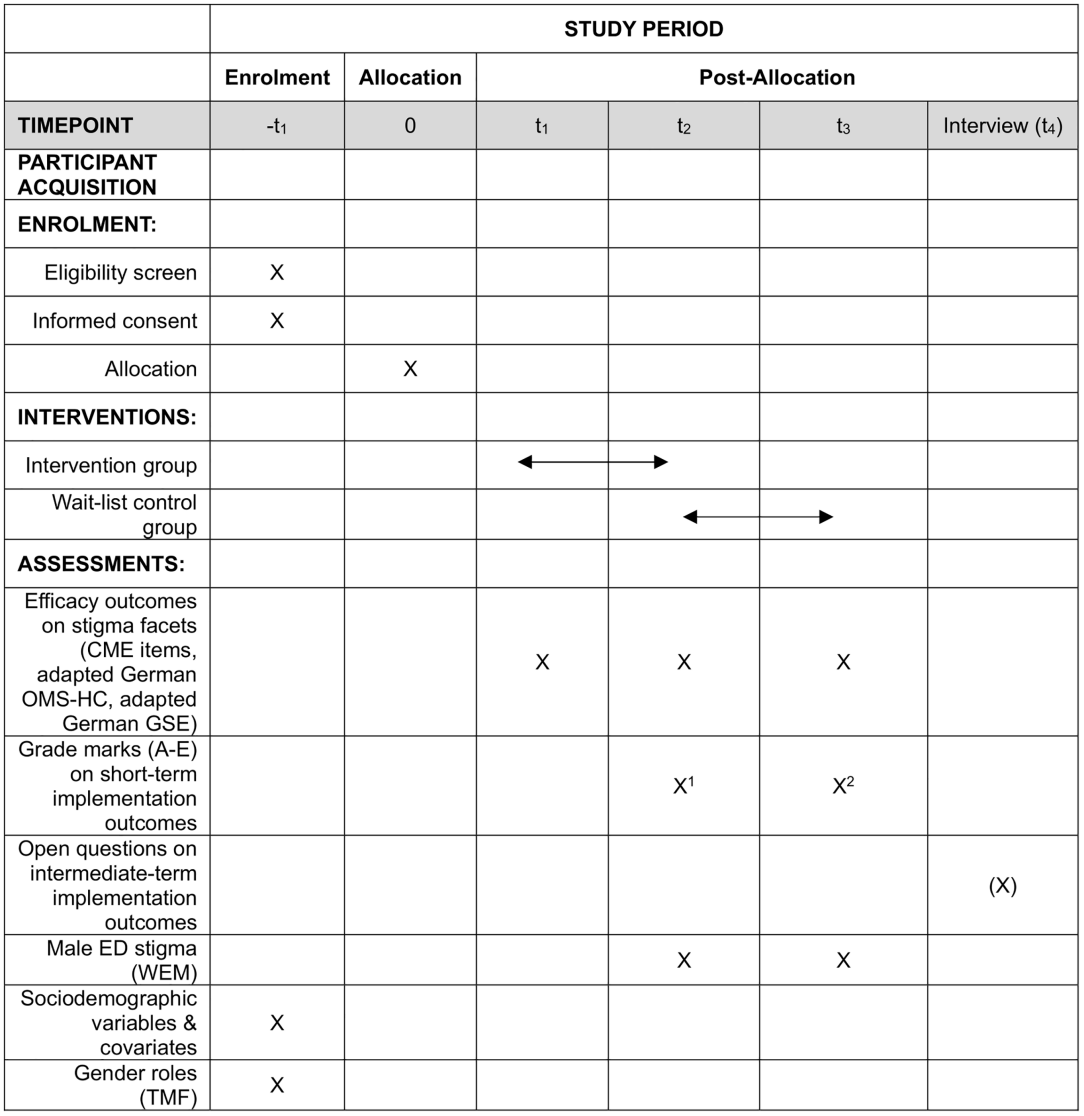
Schedule of participant acquisition, enrolment, interventions and assessments. CME = Continuing Medical Education; OMS-HC = Opening Minds Stigma Scale for Health Care Providers; GSE = General Self-Efficacy Scale; WEM = Scale on the Perception of Eating Disorders in Men; TMF = Traditional Masculinity-Femininity Scale; GP = General practitioner; ED = Eating disorder; ^1^ in intervention group; ^2^ in wait-list control group; crosses in brackets indicate optional assessments.

Individuals interested in taking part in the study can self-enroll via a webform on the project website (https://maennermitessstoerung.rub.de), which is referenced as link and QR code in leaflets and mailings. At enrollment (-t_1_), participants will answer baseline and sociodemographic questions, undergo eligibility assessment and be allocated to their respective participant group (GP or medical student) and study arm (intervention or wait-list control). Participants allocated to the wait-list control groups will be informed that they need to wait 14 days for the online training. Participants of the wait-list control groups will be meanwhile invited to a quantitative pre-participation assessment at t_1_ within the two-week waiting period, after which they will receive the personalized login credentials for the online learning platform to administer a quantitative pre-intervention assessment at t_2_ (end of the waiting period), then followed by the actual online training and a quantitative post-intervention assessment at t_3_. By contrast, participants allocated to the intervention group directly receive personalized login credentials for the learning platform at t_1_ (enrollment) and administer pre-intervention assessments before participating in the online training, followed by a quantitative post-intervention assessment at t_2_. Intervention group participants will be contacted to administer a quantitative post-participation/follow-up assessment at t_3_ another 14 days after they have completed the online training. Both groups will have 14 days to complete the training. To minimize the risk of participant dropout, participants will receive reminder emails on several time points during their participation (a reminder for completing the pre-survey 1 week after receiving the pre-survey link, a reminder for completing the online training one week after receiving login credentials and another reminder two days before termination of the 14-days participation time window, and a reminder for the post-survey 1 week after receiving the post-survey link). This procedure applies to both GPs and medical students allocated to the respective study arms. Finally, selected participants of the GP groups are invited to a qualitative telephone interview (t_4_) about four months after completion of the online training and respective quantitative assessments (see Fig 2).

### Sample size

To determine the appropriate sample size for the planned quantitative analysis (e.g., a 2x3 repeated measures ANOVA with a within-between interaction), an a priori power analysis was conducted using G*Power 3.1.9.7 software [30]. The aim was to achieve a power of.80 [31] with an alpha level of.05. A small-to-medium effect size (*f* = 0.15) as found in prior ED-related anti stigma research [15] and moderate correlation (*r* = .50) among the repeated measures were assumed, as observed in similar fields and due to practical significance considerations. The epsilon (ε) was set to 1, assuming sphericity, which is the most conservative scenario. Using these parameters, the G*Power analysis indicated that a total sample size of 74 participants is required, i.e., a minimum of 37 participants per group. Considering dropouts and cases with insufficient plausibility or quality of data, the study aims for an experimental group of approximately 50 GPs and a wait-list control group with further 50 GPs. To estimate the needs and benefits of future curricular use, the intervention will also be made accessible to approximately 50 medical students in their clinical training year. For the qualitative analyses, up to ten participants with lived experience of EDs and additional GPs for the pre-implementation focus group and panel discussion, and up to 50 GPs for the telephone-based qualitative post-implementation interviews were considered sufficient.

### Recruitment

We will advertise participation and assess participants for eligibility until achieving the target sample size. There is an estimated number of 55,000 GPs in Germany [32]. Based on an assumed response rate of up to 5% observed in similar studies [33], we targeted to contact at least 2,000 GPs in order to achieve a sample of *N* = 100 GPs in an 18-month time window. Recruitment and data collection started on July 23, 2024, following the trial’s preregistration, and are both expected to be completed until November 1, 2025. Thus, results are expected by the end of 2025.

### Assignment of interventions

Participants will be randomly assigned to either the control or intervention conditions with a 1:1 allocation as per a specified randomization schedule. Randomization will be conducted electronically. Blinding of the researcher conducting the interviews was deemed infeasible, but participants will be blinded as per the different study conditions.

### Data collection methods

All quantitative and qualitative data will be collected pseudonymously via electronic data capture tools (see below). Regarding primary outcomes, the cognitive facet of stigma (i.e., stereotypes) will be operationalized in terms of knowledge deficits regarding EDs in boys and men. We will construct and pilot a set of ten single-choice items (with five alternative answers each, from which one correct answer must be selected). The items will be collaboratively constructed based on literature search and intensive discussions within our team of experienced scientists and clinicians and the lived experience advisory board members. The global score will be calculated as the sum of correct answers over all items. The affective facet of stigma (i.e., prejudice) will be assessed using an adapted version of the validated German [25] Opening Minds Stigma Scale for Health Care Providers (OMS-HC) [26] replacing “person with a mental illness” with “men with an eating disorder”. The OMS-HC includes 15 self-report items rated on a 5-point Likert scale from 1 (*strongly disagree*) to 5 (*strongly agree*). The attitudes subscale score will serve as the primary dependent variable regarding the affective stigma facet. The behavioral facet of stigma will be assessed with three adapted items of the German version [34] of the General Self-Efficacy Scale (GSE) [27] (“I can remain calm when facing difficulties in the treatment of men with eating disorders because I can rely on my coping abilities”, “I am confident that I could deal efficiently with unexpected events in the treatment of men with eating disorders”, “When I am confronted with a man with an eating disorder, I can usually find several solutions”). Responses are made on a 4-point scale ranging from 1 (*not at all true*) to 4 (*exactly true*). An overall score will be computed as the sum of the three items.

Short-term implementation outcomes, i.e., acceptability, feasibility, appropriateness, and adoption will be assessed with one item each (“How satisfied are you with this module?”, “How do you rate the practical suitability of the module content?”, “How do you rate the relevance of the module for your day-to-day work?”, “How would you rate your willingness to incorporate the module content into your work?”). The items are all rated on 5-point Likert scales in grade marks from A to E (A = 1/*very good* to E = 5/*unsatisfactory*). These items, which are assessed both after each module and at the end of the overall training course, are complemented by two open-ended questions aimed at identifying areas for improvement (“What would you like to improve about this module?”) and capturing any additional feedback (“Is there anything else you would like to say?”).

A consensus on intermediate-term implementation outcomes will be derived from responses to open-ended questions obtained during telephone-based qualitative interviews, conducted using a semi-structured interview guideline. Intermediate-term implementation outcomes will be assessed in terms of fidelity (e.g., changes in awareness, attitudes and sensitization), penetration (e.g., modified history taking procedures, changes in the number of identified eating disorder cases in men, number of referrals to psychiatric, psychosomatic or psychotherapeutic treatment), and sustainability (e.g., changes in treatment practice such as the introduction of routine screening questions on eating behaviors, utilization of standardized diagnostic instruments, or medical case management). The interview guideline will be developed based on extensive literature search and critical discussions between the research team and advisory board members regarding both conceptualization and operationalization and considering the specific context of primary care physicians.

The secondary outcome stigma-related perceptions of EDs in men will be assessed with a previously developed set of seven items [8] based on a qualitative analysis of testimonials of men with EDs (e.g., “Eating disorders are women’s diseases”, “It is better for a man to not admit having an eating disorder”). The items are rated on a 4-point scale from 1 (*completely disagree*) to 4 (*completely agree*), and aggregated as means such that higher scores indicate higher perceived stigma of EDs in men.

Assessed sociodemographic baseline features include participants’ age, gender, sexual orientation, marital status, German language proficiency, migration background, and self-identification as part of a minority group. We further assess the job situation and characteristics of GPs (current job situation, federal state of main job activity, size of the city of main job activity, and specific setting details). Students will be asked similar questions as GPs regarding their current medical training (e.g., current semester, desired medical specialization). GPs will also be presented questions regarding their professional medical background, including duration of employment as a medical professional, specialty areas, years of experience, current position in primary care, and work experience in psychiatry. We will examine these variables for group differences and, if necessary, include them as covariates in the analyses.

For exploration, we will also at baseline assess attitudes towards mental health in GPs and medical students (e.g., practice and attitude towards the assignment of diagnoses of mental disorders, utilization of standardized diagnostic instruments, frequency of encountering patients with mental health issues, attitudes towards the referral to specialized mental health treatment facilities), and personal experiences with mental disorders and associated impairments (e.g., living with a person with an ED, having oneself received an ED diagnosis and/or treatment, living with disability). Furthermore, we will ask participants for their professional experiences with individuals with EDs at baseline.

Finally, baseline measures include the Traditional Masculinity-Femininity Scale (TMF) [29]. This scale measures self-perceived core aspects of one’s gender role-associated self-concept regarding masculinity and femininity based on traits, interests, appearance, and behaviors on two separate subscales with six items each, using two 4-point Likert scales ranging from 1 (*not at all masculine*) to 4 (*totally masculine*) and 1 (*not at all feminine*) to 4 (*totally feminine*), respectively.

To promote participant retention, participants will receive reminder emails (see section “Participant Timeline”) and a compensation for completing the survey and online intervention (i.e., monetary reimbursement for GPs, participation in the prize draw for online vouchers for medical students). Further, the survey is set up so that most questions cannot be skipped to proceed or submit the survey, with in-built reminders of unanswered items.

### Data management

Data management for quantitative data will be realized using jsPsych [35] and the Moodle learning management system’s [36] version 4.4 built-in survey modules, hosted by Ruhr-University Bochum’s IT services department. Qualitative data will be collected by means of audio recordings, ensuring privacy of the participants, since no video is recorded. The interviews will be transcribed using noScribe [37] version 0.6, a locally operated AI transcription tool based on Whisper [38], again meticulously ensuring data protection and privacy. The automated transcription will afterwards be proofread by research assistants, trained in conducting and transcribing qualitative interviews in an iterative process. All systems connected to the internet employ SSL-encryption for secure data capture and transfer. Personal data entered upon registration will be stored separately from outcome data; all outcome data (e.g., questionnaire data, interview audio files and transcripts) will be pseudonymized for the duration of the study and will be fully anonymized upon study completion.

Data will be assessed for completeness, plausibility, quality (including an attention check via the item “Have you watched the full video?” after each video/module as well as a double-participation check using the item “Have you previously completed our “Eating disorders in boys and men” course?”), and range validation.

### Data analysis

Qualitative data analyses will be conducted using MAXQDA [39] version 24.5.1. Quantitative data analyses will be performed using R [40] and SPSS [41].

Regarding quantitative data, we will conduct two sets of analyses. These will be per-protocol analyses and intention-to-treat analyses. For the per-protocol analyses, we will compute a 2 (intervention vs. wait-list control) x 3 (timepoint) mixed-measures ANOVA per each outcome and participant group, accounting for the two groups (intervention vs. wait-list control group) and the three time points of quantitative assessment. The intention-to-treat analyses will be conducted using a corresponding 2 x 3 mixed model, with by-participant random intercepts (and slopes, if justified by the data). We will also conduct contrast analysis for the 2 x 3 design (1 −1 −1 1 1–1) to directly test the predicted interaction pattern. The change from pre- to post-intervention will be estimated with a paired-samples t-test for any participant that completed the intervention. Quantitative implementation data will be aggregated descriptively and compared between groups.

The per-protocol analysis will use case-wise exclusions in case of missing data. In case of dropout/case exclusions, the ITT mixed model will be conducted with and without multiple imputation based on predictive mean matching functioning as a sensitivity analysis.

The qualitative data collected in the beginning (focus group, panel discussion) and after the intervention (during telephone-based interviews) will be subjected to systematic qualitative thematic analyses based on the Braun and Clarke method [42,43]. Coding will be performed by two independent researchers. Braun and Clarke’s [42] approach to qualitative thematic analysis follows a six-phase iterative and reflective process that evolves over time, requiring researchers to deeply engage with the data and continuously move back and forth between phases. The process begins with (1) familiarization with the data, where researchers immerse themselves in the data through repeated reading and note-taking to identify initial patterns. Next, (2) initial codes are systematically generated to capture meaningful data features. These codes are then (3) grouped into broader themes that represent meaningful relationships within the data. The identified themes (4) are then reviewed to ensure coherence and accurate representation of the dataset, resulting in further refinement and the generation of a thematic ‘map’ of the analysis. Once finalized, (5) themes are clearly named and defined. The final phase involves (6) synthesizing the findings into a cohesive report by the selection of vivid, compelling extract examples, final analysis of selected extracts, and relating back of the analysis to the research question and literature. Qualitative data will be transcribed and fully anonymized before the analysis. Regarding the telephone-based interviews, the individual subject’s report will be used as the unit of analysis. The transcripts will be coded and analyzed in MAXQDA [39] by two independent researchers as well as trained research assistants, such that each transcript will be coded by two coders independently. To establish a common understanding of the research questions and methodology, we will hold a meeting with all team members before commencing the qualitative data analysis. The findings from both the quantitative and qualitative phases will be integrated during the final data integration stage.

### Data monitoring

We will not install a data monitoring committee, as the study does not include experimental manipulations, and adverse events can be reported directly to the responsible investigators. The study information provided to participants includes the contact details of the principal investigator, along with explicit instructions to reach out at any time, if necessary.

Participation is entirely voluntary, and no harm is expected; participants will have the option to report any adverse events occurring during or after the study directly to the study investigators. Since these reports will not follow a systematic collection process, adverse events will be evaluated on a case-by-case basis in collaboration with the affected individuals. Appropriate measures for harm reduction and post-study support will be implemented as needed. Ultimately, any reported adverse events will be analyzed, quantified, and documented in scientific publications.

### Patient and public involvement

Men with EDs and GPs will be involved throughout the entire research process and design of the online training. At the beginning of the project, we will establish a lived experience council of up to ten men with EDs, occasionally joined by two GPs. The council will be involved in the selection of relevant content closely reflecting the perspectives of individuals with lived experience, in designing the intervention structure and format of delivery, as well as in its adaptation to the context of the German healthcare system. Moreover, the council will be involved in the dissemination and recruitment process by forwarding invitations and leaflets to medical professionals and students and identifying relevant access channels to the target participant group. The council will meet regularly throughout the project period, critically reflecting and discussing the progress of the project, identifying obstacles and solutions as well as interpreting the study results. Additionally, the council will be involved in writing a two-expert-perspective handbook for primary care providers on the topic of EDs in boys and men.

### Ethics and dissemination

#### Research ethics approval.

The study is based on the guidelines of Good Scientific Practice of the German Research Foundation and was approved by the Ethics Committee of the Ruhr-University Bochum’s Medical Faculty at Campus East-Westphalia (AZ 2023−1106, November 7, 2023).

#### Consent.

Participants of both the advisory board and the actual online training intervention throughout the different phases are informed and educated about the procedure and study goals prior to data collection (for consent forms and written study information, see **Supplement**). Written informed consent, as defined by the Declaration of Helsinki, is a prerequisite for study participation and will be obtained from all participants prior to their enrollment. All participants will receive a copy of their submitted consent forms at their disposal.

#### Confidentiality and dissemination policy.

All data relevant to the study is pseudonymized, digitally transferred to the statistical software, and stored on a password-protected server that is only accessible to authorized employees via secured network connections. Data will only be transferred in pseudonymized form. Pseudonymized data will be stored separately from consent forms. All data collected as part of this study will be deleted after 10 years. Participants will also be informed of their rights under the European General Data Protection Regulation (as of 25.05.2018) (GDPR) and the Data Protection Act of North Rhine-Westphalia (DSG NRW) (as of 09.03.2021).

Study reporting will adhere to the Checklist for Evaluation-Specific Standards (CHESS) [44]. Following principles for open science (e.g., the German Psychological Society’s recommendations on Open Science Practices), the study protocol and key findings will be published openly, along with anonymized, group-level aggregated data. Additionally, the statistical code will be provided as supplementary material in publications documenting the study results.

### Protocol amendments

Any deviations from the original study design will be documented in the journal(s) in which the results will be published.

## Discussion

The aim of the proposed iSMEsH training intervention is to sensitize GPs in primary care in Germany to the occurrence and manifestation of EDs in men, impart knowledge and specific skills, and by this combat the stigmatization of EDs as “women’s diseases”. The proposed project involves the development, dissemination, and evaluation of the online training. The proposed study examines the efficacy and implementation of the intervention with a mixed-methods evaluation design. The study includes a pre-implementation stage with qualitative and quantitative data collection, an implementation stage with a randomized, wait-list controlled evaluation of the training intervention, and a post-implementation stage with qualitative interviews.

### Strengths and limitations

In developing this study protocol, we have identified both methodological strengths and potential limitations. One of the key strengths of this study is its comprehensive approach to addressing multiple facets of stigmatization. By targeting primary care physicians, the intervention aims to improve the detection and treatment of EDs in men, who are often underrepresented in ED research and care. The involvement of individuals with lived experience and public involvement ensures that the intervention closely reflects the needs and perspectives of those affected. Additionally, the sequential mixed-methods evaluation design promotes a broad understanding of the efficacy and implementation outcomes of the intervention. Despite its strengths, the study has some limitations. While it focuses on short- and intermediate-term implementation outcomes, long-term implementation outcomes are not assessed. This restricts the ability to draw conclusions regarding the long-term effects of the intervention. Additionally, the study’s reliance on self-reported data and its limitation to the specific context of the German healthcare system need to be considered regarding the generalizability of the findings.

## Conclusions

In conclusion, this study addresses stigmatization as a prominent barrier to the treatment of men with EDs. The iSMEsH intervention has the potential to improve the detection and treatment of EDs in men, i.e., reducing stigmatization of men with EDs, by sensitizing GPs and equipping them with the necessary knowledge and skills.

## Supporting information

S1 Table SPIRIT checklist. (DOCX)

## Abbreviations

ED: Eating Disorder
GP: General Practitioner
CME: Continuing Medical Education
OMS-HC: Opening Minds Stigma Scale for Health Care Providers
GSE: General Self-Efficacy Scale
TMF: Traditional Masculinity-Femininity Scale
iSMEsH: Intervention Against the Stigmatization of Men with Eating Disorders in Primary Care
